# Scan–rescan reproducibility of segmental aortic wall shear stress as assessed by phase-specific segmentation with 4D flow MRI in healthy volunteers

**DOI:** 10.1007/s10334-018-0688-6

**Published:** 2018-05-26

**Authors:** Roel L. F. van der Palen, Arno A. W. Roest, Pieter J. van den Boogaard, Albert de Roos, Nico A. Blom, Jos J. M. Westenberg

**Affiliations:** 1 Division of Pediatric Cardiology, Department of Pediatrics, Leiden University Medical Center, Albinusdreef 2, 2333 ZA Leiden, The Netherlands; 2 Department of Radiology, Leiden University Medical Center, Albinusdreef 2, 2333 ZA Leiden, The Netherlands

**Keywords:** 4D flow MRI, Wall shear stress, Aorta, Aortopathy

## Abstract

**Objective:**

The aim was to investigate scan–rescan reproducibility and observer variability of segmental aortic 3D systolic wall shear stress (WSS) by phase-specific segmentation with 4D flow MRI in healthy volunteers.

**Materials and methods:**

Ten healthy volunteers (age 26.5 ± 2.6 years) underwent aortic 4D flow MRI twice. Maximum 3D systolic WSS (WSSmax) and mean 3D systolic WSS (WSSmean) for five thoracic aortic segments over five systolic cardiac phases by phase-specific segmentations were calculated. Scan–rescan analysis and observer reproducibility analysis were performed.

**Results:**

Scan–rescan data showed overall good reproducibility for WSSmean (coefficient of variation, COV 10–15%) with moderate-to-strong intraclass correlation coefficient (ICC 0.63–0.89). The variability in WSSmax was high (COV 16–31%) with moderate-to-good ICC (0.55–0.79) for different aortic segments. Intra- and interobserver reproducibility was good-to-excellent for regional aortic WSSmax (ICC ≥ 0.78; COV ≤ 17%) and strong-to-excellent for WSSmean (ICC ≥ 0.86; COV ≤ 11%). In general, ascending aortic segments showed more WSSmax/WSSmean variability compared to aortic arch or descending aortic segments for scan–rescan, intraobserver and interobserver comparison.

**Conclusions:**

Scan–rescan reproducibility was good for WSSmean and moderate for WSSmax for all thoracic aortic segments over multiple systolic phases in healthy volunteers. Intra/interobserver reproducibility for segmental WSS assessment was good-to-excellent. Variability of WSSmax is higher and should be taken into account in case of individual follow-up or in comparative rest–stress studies to avoid misinterpretation.

**Electronic supplementary material:**

The online version of this article (10.1007/s10334-018-0688-6) contains supplementary material, which is available to authorized users.

## Introduction

Four-dimensional flow magnetic resonance imaging (4D flow MRI) is a non-invasive imaging method to investigate in vivo cardiovascular flow and is used to better understand cardiovascular physiology and pathophysiology. Besides simple measures of flow [1–3], this technique allows for quantification of more comprehensive hemodynamic parameters, such as wall shear stress (WSS) [4, 5]. WSS is defined as the viscous shear force of flowing blood acting tangentially to the vessel wall and can be estimated in both small and large vessels. Alterations in WSS within the aorta have been associated with vascular wall remodelling and dysfunction [6] and this hemodynamic parameter is therefore of interest in patients with aortopathy.

Aortic WSS can be calculated from 4D flow MRI data based on 2D cross-sectional planes at specific locations along the vessel wall [7] or by volumetric 3D WSS computational algorithms [8]. It is known that several acquisition and post-processing factors may influence the estimation of WSS, e.g., spatial resolution, settings of encoding velocity (VENC parameter), signal-to-noise ratio and segmentation inaccuracies [9, 10]. Estimation of WSS from 4D flow MRI data necessitates analysis of velocity data within a geometric representation of the 3D vascular structure of interest through segmentation (i.e., creating a cast of the vessel of interest) [11]. Currently, thoracic aorta segmentations in most studies are based on phase contrast MR angiograms averaged over all cardiac time frames or manually drawn for every cardiac time frame in 2D planes. Using these approaches, accurate WSS reproducibility has been proven in healthy volunteers [7, 12]. However, due to movement of the aorta during the cardiac cycle, aortic compliance and peak systolic time-differences of the propagating blood flow wave front along the thoracic aorta, calculations of WSS throughout the systolic cardiac cycle from a time frame averaged aortic cast could lead to an incorrect assessment of true aortic border velocities for aortic segments along the thoracic aorta.

Therefore, the aim of the study was to assess scan–rescan reproducibility and observer variability of segmental aortic 3D WSS based on semi-automatic, systolic phase-specific 3D aortic segmentation from 4D flow MRI in healthy volunteers. To put the reproducibility analysis of segmental WSS approach into clinical perspective, aortic WSS analyses of two patient cases with congenital aortic abnormalities were compared to the healthy volunteers.

## Materials and methods

### Study population

The study protocol was approved by the Medical Ethics Committee of the Leiden University Medical Center and informed consent was obtained from all participants. Ten healthy volunteers (age 26.5 ± 2.6 years) without history of cardiovascular disease were included. All subjects underwent a cardiovascular MR examination including aortic 4D flow MRI. The same scanning protocol was performed twice (i.e., scan and rescan) in the same session with a 10-min break between the scans and repositioning and replanning for every volunteer.

### Cardiovascular MR imaging acquisition and 4D flow data processing

Cardiovascular MR imaging, including aortic 4D flow imaging, was performed on a 3.0 T scanner (Ingenia, Philips Medical Systems, The Netherlands with Software Stream 4.1.3.0) with FlexCoverage Posterior coil in the table top with a dStream Torso coil, providing up to 32 coil elements for signal reception. Aortic 4D flow MRI was acquired during free breathing using respiratory navigator gating based on hemidiaphragm excursion and retrospective ECG gating with full 3D coverage of the thoracic aorta in an oblique sagittal orientation. 4D flow MRI sequence parameters were as follows: velocity-encoding of 200 cm/s in three directions, spatial resolution: 2.5 × 2.5 × 2.5 mm^3^, temporal resolution: 35.1–36.5 ms, echo time/repetition time: 2.5–2.7 ms/4.4–4.6 ms, flip angle: 10°, field of view: 350 × 250 × 75 mm, TFE factor: 2, sensitivity encoding (SENSE) factor 2.5 in anterior–posterior direction. Concomitant gradient correction and local phase correction was performed from standard available scanner software. Acquisition time was on average 12 min.

4D flow MRI data was imported into the commercially available software program CAAS MR 4D flow v1.1 (Pie Medical Imaging BV, Maastricht, The Netherlands). Additional phase offset correction and anti-aliasing was performed in the CAAS MR software package.

The peak systolic cardiac phase was automatically detected by the CAAS MR Flow software program by identification of the cardiac phase with the highest variance in the 3D velocity dataset. For this phase, the segmentation was initialized by manual placing two delimiter points (start and endpoint): one in the subaortic region of the left ventricular outflow tract (start point) and one in the descending aorta (end point), at the same level as the start point. A phase-specific 3D aortic volume was automatically segmented for this peak systolic phase plus two consecutive phases before and two phases after this peak systolic phase (i.e., peak systolic phase − 2, peak systolic phase − 1, peak systolic phase, peak systolic phase + 1 and peak systolic phase + 2.) The 3D segmentation uses a deformable model algorithm [13] that recursively optimizes the location of the surface towards the vessel luminal boundary based on image gradients, extracted from the appropriate phase within the 4D flow MRI data, while simultaneously maintaining local smoothness of the 3D segmented surface. Manual delineation of the vessel lumen boundary was applied with the available adaptation tool from the software in case of segmentation incorrectness.

### Segmental WSS assessment

**Fig. 1:**
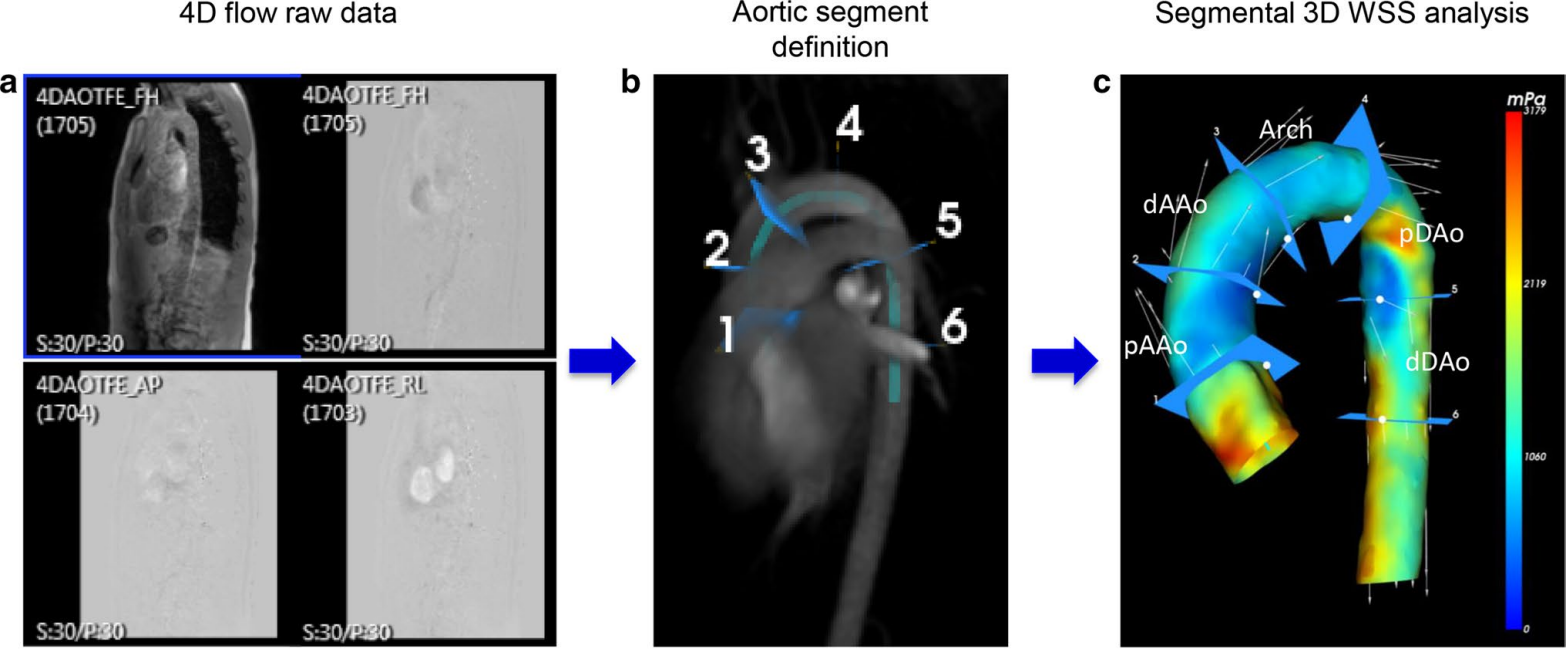
Aortic 4D flow MRI processing and analysis. **a** 4D flow MRI raw data including anatomical and flow data. **b** Automatic segmentation of 3D aortic volume after manually defining start and endpoint of the thoracic aorta and aortic segment definition. **c** 3D color-coded aortic segmentation representing WSSmax distribution for one systolic cardiac phase

The surface of the 3D segmented aorta consisted of wall points and for each wall point the 3D systolic WSS vector was calculated based on a quadratic approximation of the axial velocity profile perpendicular to the surface of the 3D segmented aorta. For the regional assessment of maximum aortic 3D systolic WSS (WSSmax) and mean 3D systolic WSS (WSSmean) the thoracic aorta was manually divided into five aortic segments based on anatomic landmarks (Fig. 1). This aortic subdivision was applied to each of the five phase-specific segmentations. Segment 1: proximal ascending aorta (pAAo, from the sinotubular junction to the mid-ascending aorta); segment 2: distal ascending aorta (dAAo, from the mid-ascending aorta to the origin of the innominate artery), segment 3: aortic arch (Arch, from the origin of the innominate artery until the left subclavian artery), segment 4: proximal descending aorta (pDAo, beyond the left subclavian artery to the mid-descending thoracic aorta); and segment 5: distal descending aorta (dDAo, from the mid-descending thoracic aorta to the descending aorta at the level of the aortic valve). Of note, supra-aortic arch branches were excluded from segmental WSS measurements. WSSmax was defined as the maximum WSS vector of all wall points within the defined aortic segment. WSSmean was defined as the average of all WSS vectors of all wall points within the defined aortic segment.

To assess scan–rescan reproducibility, the two consecutively acquired 4D flow MRI scans for each healthy volunteer were analyzed and compared: segmental aortic WSS analysis (i.e., including segmentation and adaptation, and subsequent division of the aorta into five segments) for five systolic cardiac phases (peak systolic phase ± 2 phases) was performed for the first and second 4D flow MRI scan data (i.e., scan and rescan) by a single observer (*RP*) in all volunteers. All data was presented blinded to the observers who analyzed the data in a random order. To assess intraobserver variability, segmental aortic WSS analysis was performed twice for five systolic cardiac phases (peak systolic phase ± 2 phases) from the first acquired 4D flow MRI scan by a single observer (*RP*) in all volunteers. To assess interobserver variability, segmental aortic WSS analysis was performed for five systolic cardiac phases (peak systolic phase ± 2 phases) from the first acquired 4D flow MRI scan by two observers (*RP* and *PB*) in all volunteers. The analysis took approximately 18 min per systolic phase per volunteer.

To put the reproducibility analysis of segmental WSS approach into clinical perspective, two patient cases with congenital aortic abnormalities were included: Case 1. A 13-year-old male patient with non-stenotic residual narrow proximal descending aortic segment after surgical correction for aortic coarctation at the age of 1 month; and Case 2. A 17-year-old male patient with ascending aortic dilation after arterial switch operation for transposition of the great arteries prior to replacement of the proximal ascending aorta. Cardiovascular MR imaging, including 4D flow MRI and post-processing, was performed in these cases according to above described methods. An aortic segmentation was made and WSS calculations (WSSmax and WSSmean) were performed for the peak systolic cardiac phase for the aortic segments of interest for the two patients. For patient case 1 this included the pDAo segment, for patient case 2 the ascending aortic segments (pAAo and dAAo).

### Statistical analysis

Statistical analysis was performed using IBM SPSS Statistics 20.0 (SPSS Inc., Chicago, IL, USA). Normality assumptions for all variables were assessed using a Shapiro–Wilk test. All continuous parameters were expressed as mean ± standard deviation (SD). Paired *t* test was used to determine heart rate differences and differences in time-points of the systolic cardiac phases between the scan and rescan data. To assess the degree of agreement for scan–rescan and intra- and interobserver comparison, mean differences and limits of agreement (± 2 SD of mean difference) of WSSmean and WSSmax for the different aortic segments from five consecutive cardiac systolic phases were calculated by Bland–Altman analysis [14]. Correlation between measurements of the scan and rescan as well as for intra- and interobserver comparison was tested by the Spearman correlation coefficient (*r*). To evaluate the extent of variability in relation to the mean, the coefficient of variation (COV) was calculated as: standard deviation of mean difference for the WSS parameter (WSSmean or WSSmax) between scan–rescan divided by the group average of that WSS parameter for each aortic segment. Reliability between two scans was assessed by the intra-class correlation (ICC) coefficient. Correlation and agreement were classified as follows: *r* or ICC ≥ 0.95: excellent, 0.94–0.85: strong, 0.84–0.70: good, 0.69–0.50: moderate, < 0.50: poor. The COV was classified as: low (≤ 10%), intermediate (11–20%), high (21–30%) and very high (≥ 31%). A *P* value < 0.05 was considered statistically significant.

## Results

Baseline characteristics of the volunteers are shown in Table 1. Average heart rate (HR) was similar in the volunteers for the scan and rescan (60.8 ± 7.7 vs 61.6 ± 6.0 bpm, *P *= 0.65). Average time-points of the five systolic phases (ms) throughout the cardiac cycle were not significantly different between the scan and rescan data from the volunteers (Table 1).

**Table 1:** Baseline characteristics

	Volunteers	Scan	Rescan	*P* value*
*N*	10			
Male (%)	5 (50%)			
Age (years)	26.5 ± 2.6			
Height (cm)	175.6 ± 6.6			
Weight (kg)	68.3 ± 12.7			
BSA (m^2^)	1.8 ± 0.2			
Heart rate (bpm)		61 ± 8	62 ± 6	0.65
Time-points throughout cardiac cycle (ms)				
Peak systolic phase − 2		104.1 ± 19.2	103.4 ± 19.0	0.91
Peak systolic phase − 1		136.8 ± 18.4	135.4 ± 18.1	0.86
Peak systolic phase		169.0 ± 17.5	167.5 ± 17.6	0.85
Peak systolic phase + 1		201.5 ± 17.6	199.9 ± 17.2	0.83
Peak systolic phase + 2		233.6 ± 17.6	232.0 ± 17.1	0.83

Data presented as mean ± standard deviation

*BSA* body surface area, *bpm* beats per minute, *ms* milliseconds

*Paired sample *t* test

### Scan–rescan reproducibility

**Fig. 2:**
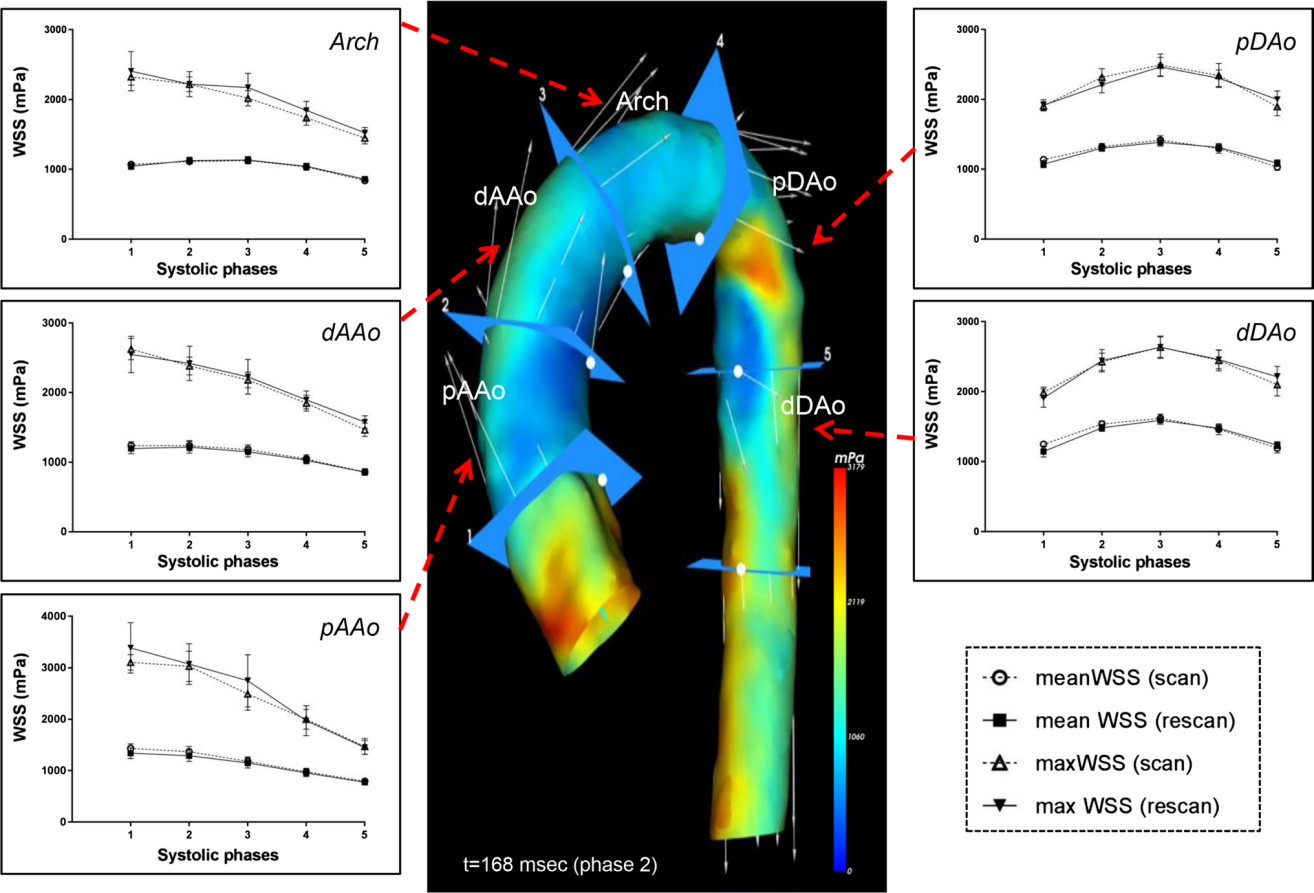
Mean and maximum systolic WSS over five systolic cardiac phases around peak systole (peak systolic phase ± 2 phases) across the five thoracic aortic segments for all volunteers. Error bars represent SEM

Figure 2 shows the group averaged WSS measurements over five systolic cardiac phases (peak systolic phase ± 2 phases) across the five thoracic aortic segments for all volunteers for the scan and rescan. The average WSSmean shows very similar values for scan and rescan and both average WSSmean and average WSSmax closely follow a similar trend during the five systolic cardiac phases.

**Fig. 3:**
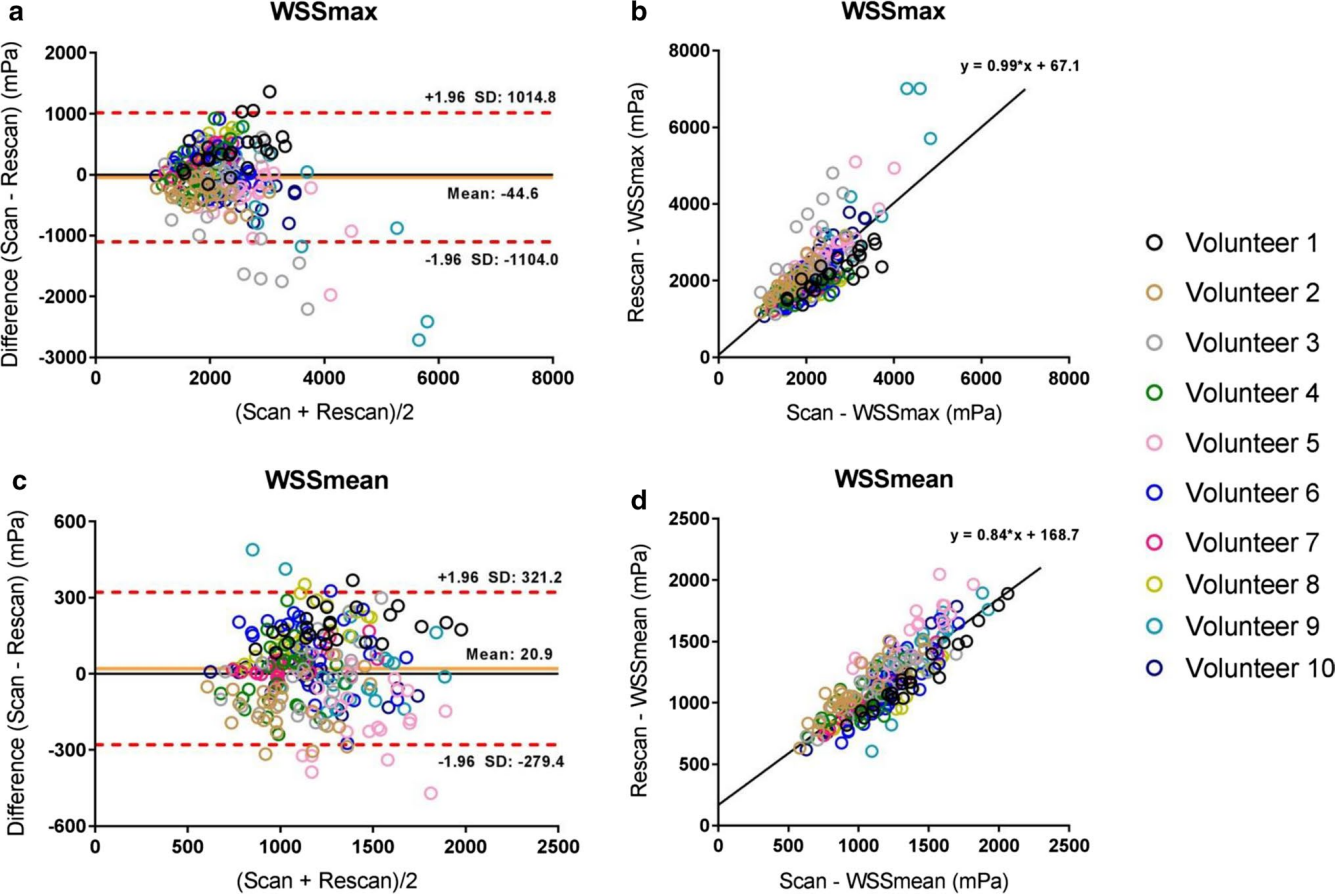
Bland–Altman and correlation plots for scan–rescan analysis for the WSSmax (**a**, **b**) and WSSmean (**c**, **d**) for five systolic cardiac phases (peak systolic phase ± 2 phases)

Results from the scan–rescan reproducibility analysis for regional aortic WSSmean and WSSmax are presented in Table 2. For all thoracic aortic segments, moderate to good ICCs and Spearman correlations were found for WSSmax and overall good to strong ICCs with good to strong correlation for WSSmean. COV of segmental systolic WSS measurements between the scan and rescan were 16–31% for WSSmax and 10–15% for WSSmean. For the WSSmax, this degree of variation was higher for the ascending aortic segments compared to the descending aortic segments, on average 26–31 vs 16–18%, respectively. Bland–Altman plots and correlation diagrams for scan–rescan analysis for WSSmax and WSSmean of all aortic segments during five systolic cardiac phases are depicted in Fig. 3.

**Table 2:** Segmental WSS analysis (WSSmax and WSSmean) scan vs rescan

	WSSmax (mPa)						WSSmean (mPa)					
	Bland–Altman		COV (%)	Correlation*		ICC	Bland–Altman		COV (%)	Correlation*		ICC
	Mean difference (mPa)	Limits of agreement (± 2*σ*) (mPa)		*r*	*P*		Mean difference (mPa)	Limits of agreement (± 2*σ*) (mPa)		*r*	*P*	
Proximal AAo	− 107	1536	31	0.86	< 0.001	0.79	50	323	14	0.90	< 0.001	0.89
Distal AAo	− 32	1115	26	0.68	< 0.001	0.63	23	229	10	0.89	< 0.001	0.88
Aortic arch	− 84	1066	27	0.71	< 0.001	0.55	− 4	260	13	0.72	< 0.001	0.70
Proximal DAo	12	700	16	0.68	< 0.001	0.69	10	359	15	0.64	< 0.001	0.63
Distal DAo	− 12	815	18	0.65	< 0.001	0.66	26	342	12	0.79	< 0.001	0.76

*AAo* ascending aorta, *DAo* descending aorta, *COV* coefficient of variation, *ICC* intraclass correlation coefficient

*Spearman correlation coefficient

### Intraobserver analysis

Table 3 and Supplementary Tables 1–4 shows the results of the intraobserver reproducibility analysis. Both WSSmax and WSSmean showed overall good to excellent correlation for the different aortic segments (WSSmax: *r* = 0.66–0.99, *P* < 0.001–0.038); WSSmean: *r* = 0.82–1.00, *P* < 0.001–0.004) with overall strong to excellent ICC and low COV indicating excellent reproducibility (range COV WSSmax 1–14%; range COV WSSmean 1–7%) (Supplementary Tables 1–4). Larger variability for WSS measurements of the ascending aortic segments was shown compared to the measurements of the aortic arch and descending aortic segments, more pronounced for the WSSmax than for WSSmean.

**Table 3:** Intraobserver variability of segmental WSS analysis of the *peak systolic cardiac phase* from the scan exams

	WSSmax (mPa)						WSSmean (mPa)					
	Bland–Altman		COV (%)	Correlation*		ICC	Bland–Altman		COV (%)	Correlation*		ICC
	Mean difference (mPa)	Limits of agreement (± 2*σ*) (mPa)		*r*	*P*		Mean difference (mPa)	Limits of agreement (± 2*σ*) (mPa)		*r*	*P*	
Proximal AAo	47.4	628.3	13	0.96	<0.001	0.94	21.1	100.6	5	0.99	< 0.001	0.98
Distal AAo	87.4	369.6	9	0.78	0.008	0.87	17.8	48.3	2	0.95	< 0.001	0.99
Aortic arch	69.1	157.4	4	0.95	< 0.001	0.97	14.9	48.3	2	0.99	< 0.001	0.99
Proximal DAo	− 1.6	139.9	3	0.99	< 0.001	0.99	12.3	68.7	2	1.00	< 0.001	0.98
Distal DAo	− 51.7	495.9	8	0.93	< 0.001	0.89	− 16.3	82.2	3	0.98	< 0.001	0.98

*AAo* ascending aorta, *DAo* descending aorta, *COV* coefficient of variation, *ICC* intraclass correlation coefficient

*Spearman correlation coefficient

### Interobserver analysis

Results from the interobserver reproducibility analysis are depicted in Table 4 and Supplementary Tables 5–8. WSSmax between the two observers shows overall good to excellent correlation (*r* = 0.77–0.98, *P* < 0.001–0.009) with strong to excellent ICC and low to intermediate COV (2–17%), indicating good reproducibility. WSSmean between the two observers shows good to excellent correlation (*r* = 0.76–1.00, *P* < 0.001–0.011) with overall excellent ICC and low COV (1–11%), indicating excellent reproducibility (Supplementary Tables 5–8). Least variability in WSS parameters is seen in the aortic arch and descending aortic segments.

**Table 4:** Interobserver variability of segmental WSS analysis of the *peak systolic cardiac phase* from the scan exams

	WSSmax (mPa)						WSSmean (mPa)					
	Bland–Altman		COV (%)	Correlation*		ICC	Bland-Altman		COV (%)	Correlation*		ICC
	Mean difference (mPa)	Limits of agreement (± 2*σ*) (mPa)		*r*	*P*		Mean difference (mPa)	Limits of agreement (± 2*σ*) (mPa)		*r*	*P*	
Proximal AAo	− 182.4	924.7	17	0.81	0.005	0.89	− 59.8	197.1	8	0.82	0.004	0.94
Distal AAo	− 27.3	466.3	11	0.81	0.005	0.85	11.2	35.5	2	0.96	< 0.001	1.00
Aortic arch	58.8	234.7	6	0.94	< 0.001	0.93	26.3	40.3	2	0.99	< 0.001	0.99
Proximal DAo	− 44.6	229.7	5	0.98	< 0.001	0.97	1.6	103.7	4	0.96	< 0.001	0.96
Distal DAo	− 155.0	418.1	8	0.93	< 0.001	0.90	− 22.7	94.1	3	0.96	< 0.001	0.97

*AAo* ascending aorta, *DAo* descending aorta, *COV* coefficient of variation, *ICC* intraclass correlation coefficient

*Spearman correlation coefficient

### Clinical application of segmental WSS assessment—patient cases

**Fig. 4:**
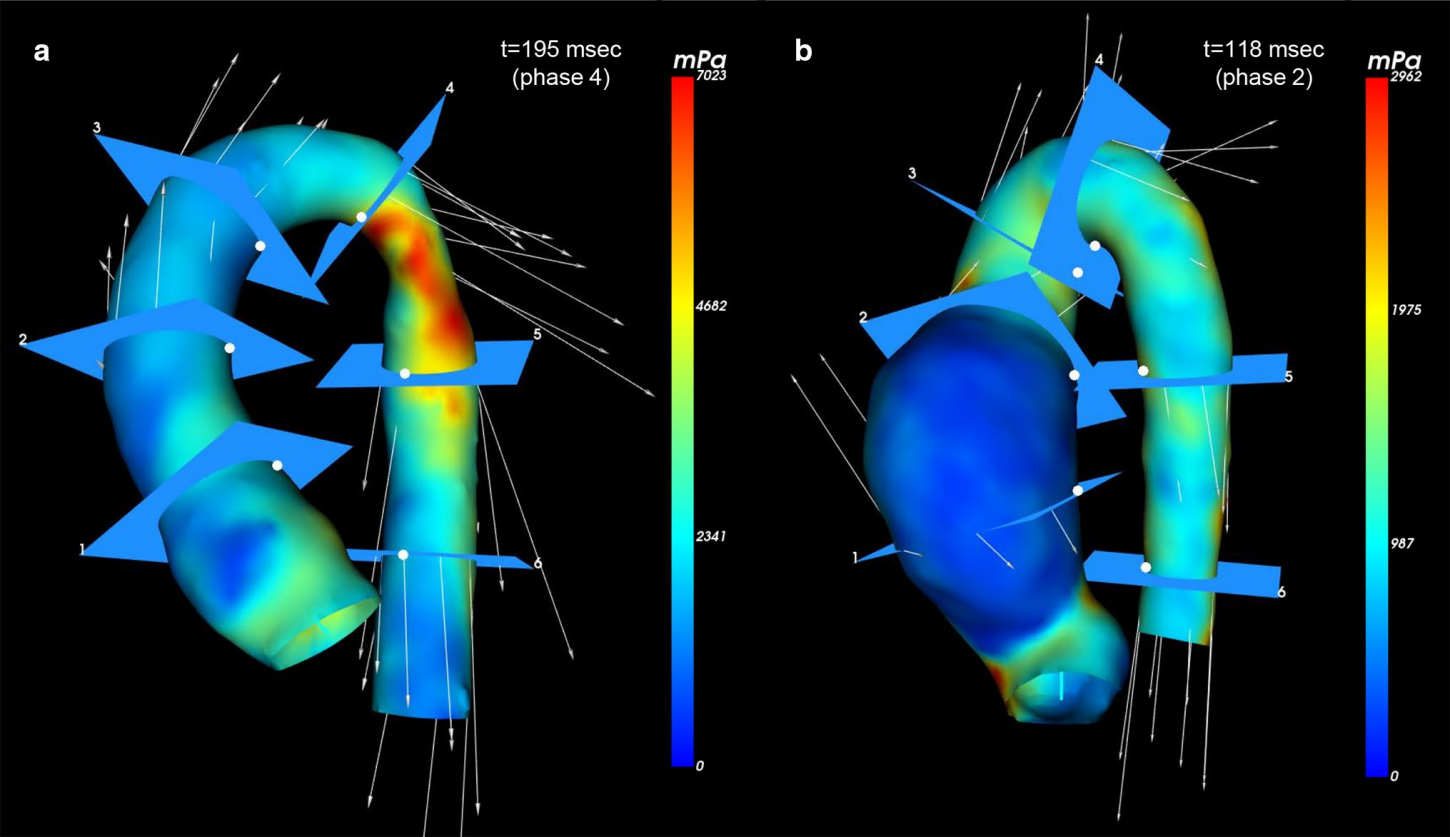
Color-coded aortic model representing WSSmax distribution from two patients with congenital heart disease involving the aorta. **a** Aortic model from a 13-year-old male patient with non-stenotic residual narrow proximal descending aortic segment after surgical correction for aortic coarctation. **b** Aortic model from a 17-year-old male patient with severe dilation of the proximal ascending aorta after neonatal arterial switch operation for transposition of the great arteries

Figure 4 shows two examples of aortic WSS assessment by 4D flow MRI in patients with congenital heart disease involving the aorta. Panel 4A shows the color-coded aortic model of the WSSmax distribution from a 13-year-old patient after neonatal aortic coarctation repair with a residual non-stenotic narrow proximal descending aortic segment (patient 1). Peak systolic WSSmax and WSSmean in the post-coarctation region were 7023 and 3444 mPa, respectively. These WSS values are high and far above the upper 95% confidence limit compared to the average WSS measures in the proximal descending aortic segment of the ten healthy volunteers (scan/rescan: WSSmax 2492 ± 497/2466 ± 431 mPa; WSSmean 1418 ± 199/1386 ± 180 mPa) (Fig. 2).

Panel 4B shows the color-coded aortic model of WSSmax distribution from a 17-year-old male patient after neonatal arterial switch operation for transposition of the great arteries (TGA) with severely dilated proximal ascending aortic segment (52 mm) prior to ascending aorta replacement (patient 2). Regional peak systolic WSS values of the proximal ascending aortic segment in this TGA patient: WSSmax 2564 mPa; WSSmean 842 mPa. These WSS values were lower compared to the average WSS measures found in the proximal ascending aortic segment in the 10 healthy volunteers (scan/rescan: WSSmax 3106 ± 479/3385 ± 1553 mPa and WSSmean 1432 ± 273/1340 ± 335 mPa) (Fig. 2).

## Discussion

The results of this study demonstrate reproducibility of segmental aortic systolic 3D WSS measures in the thoracic aorta of healthy volunteers, by phase-specific 4D flow MRI segmentation. Scan–rescan reproducibility was good for WSSmean for all thoracic aortic regions but scan–rescan reproducibility for WSSmax was moderate with higher variability up to 31% in the proximal ascending aorta. The intraobserver and interobserver reproducibility for segmental systolic WSS analysis of WSSmax and WSSmean was good to excellent. In general, the ascending aortic segments showed more variability in WSSmax and WSSmean measurements compared to aortic arch or descending aortic segments for scan–rescan, intraobserver and interobserver comparison.

Deriving accurate WSS from 4D flow MR velocity data remains challenging and delineation of the aortic wall influences the accuracy of this WSS estimation [9, 10]. Most of the previously reported studies on 3D WSS assessment make use of segmentations based on phase contrast MR angiograms averaged over all cardiac time frames to assess aortic WSS over the entire systolic cardiac cycle [12, 15–17]. In this study, we used a phase-specific segmentation approach for 3D WSS calculations from velocity data for five consecutive systolic cardiac phases. A major advantage of this phase-specific segmentation approach is that it will approximate the aortic wall of the thoracic aorta more accurate in order to better estimate true border WSS. Due to the aortic compliance and movement of the aorta, calculations from a segmentation from averaged phase contrast MR angiograms over all cardiac time frames could lead to an incorrect assessment of WSS along the thoracic aortic wall. Furthermore, the phase-specific segmentations enable identification of peak systolic WSS for each aortic segment along the thoracic aorta, which for every aortic segment is reached at a different time frame throughout systole.

The ability to measure advanced aortic hemodynamic parameters make 4D flow MRI potentially valuable for patients with different entities of aortopathy: to assess disease severity, to better anticipate disease progression and potentially stratify patients at risk for adverse events. However, knowledge of scan–rescan variability for regional WSS measures is essential to study interactions between hemodynamic parameters and aortic geometry and to judge whether hemodynamic changes in patients over time represent true (patho)physiological changes.

Few studies have investigated scan–rescan reproducibility and/or observer variability of aortic WSS measurements in vivo [7, 12, 18]. Similar to our findings, these studies reported good scan–rescan reproducibility for WSSmean measures with relatively higher scan–rescan variability for WSSmax measures. However, these WSS reproducibility studies used different approaches for WSS estimation varying from a time-resolved 2D planar WSS quantification method with manual 2D aortic segmentation [7] to a volumetric 3D WSS assessment from an aortic segmentation from phase contrast MRAs averaged over all cardiac time frames [12]. Furthermore, different time intervals between first and second scan in these studies were chosen compared to this study and varied from 2 to 4 weeks [12] to 1 year [7]. Moreover, in the study by van Ooij et al. [12] anisotropic voxels of different sizes for different subjects were used, that might have resulted in accuracy problems of WSS estimations between subjects. Our study extends the WSS reproducibility knowledge with its phase-specific, semi-automated 3D WSS segmentation approach with segmental aortic WSS assessment from a non-contrast enhanced 4D flow MRI data acquisition. The good to excellent intra- and interobserver reproducibility from this study is in accordance with these reported studies [7, 12] and a study by Bieging et al. [18], in which observer reproducibility was evaluated by a time-resolved volumetric 3D aortic WSS approach for contrast-enhanced 4D flow MRI.

In general, ascending aortic segments showed more variability in WSSmax and WSSmean compared to aortic arch or descending aortic segments for scan–rescan, intra- and interobserver comparison. Operator and subject dependent factors may have contributed to the differences found in WSS reproducibility for different aortic segments. First, the sub-optimal anatomic information within the 4D flow dataset hampers the exact manual determination of the aortic segments based on anatomic landmarks; especially identifying the sinotubular junction to determine the proximal ascending aortic segment proved to be challenging. Second, variability due to movement and systolic longitudinal stretch of the ascending aorta cannot entirely been ruled out as an influencing factor, despite the phase-specific segmentation has been applied. Heart-beat related motion and longitudinal stretch during systole has been reported to be more present in the ascending aorta compared to the aortic arch and descending aorta [19, 20]. Third, small individual variations in heart rate might have affected WSS differences between scan and rescan, although the averaged heart rate of each individual volunteer was not significantly different between the consecutive scans. Greater variability was found in WSSmax for both scan–rescan, but in general WSSmax values are more subject to noise than WSSmean values. Furthermore, the VENC setting affects the velocity-to-noise ratio and therefore may have influence on the WSS calculations in the low velocity range near the aortic wall. In this study, VENC was 200 cm/s, which was chosen with respect to the anticipated peak velocity in the full aorta, in order to avoid phase wrapping. However, the VENC setting was identical between the scan and rescan and differences due to the velocity sensitivity between both scans are therefore unlikely.

In patients with aortopathy, the aorta can be regionally affected or being entirely involved in the disease, depending on its expression. Aortic WSS disturbances have been shown to strongly correlate with molecular and architectural medial aortic wall alterations [6] or show a direct relation with its regional aortic geometry [16, 21]. The regional WSS analysis by phase-specific segmentation models in the presented patient cases provides its clinical applicability in locally diseased aortas, as the WSS values in patient 1 fall far beyond the confidence intervals of the healthy controls and the WSS values in patient 2 are considerably lower than these of the healthy controls with this WSS approach. Using this method, it shows that segment-specific aortic WSS estimation is discriminative and emphasizes the importance of accurate knowledge of WSS reproducibility and consistency across different aortic segments for each applied WSS method.

A limitation of this study is that the study consisted of only ten healthy volunteers with a relatively small age range. A larger number of volunteers with a larger variation of age could have provided more information about the robustness of this method over ages. The study did not include patients with aortopathy for a scan–rescan comparison, which could have provided more insight in the reproducibility of this method for clinical use in patients with aortic disease. The spatial and temporal resolution was similar for the scan-and rescan and both were performed without use of contrast agents. The latter could be considered as a limitation as the use of contrast agents might have increased our signal-to-noise ratio and therefore our reproducibility. However, in the light of the recent discussions on the use of contrast agents in cardiovascular MR, we tested the robustness of the WSS assessment method with 4D flow MRI without contrast [22]. The scan and rescan for every volunteer in this study was performed on the same day, which is the most ideal circumstance for a reproducibility analysis, as the volunteers were in the same cardiovascular, neurohormonal and mental status, at the same scanner, and at the similar moment of the day. Repeated scans on separate days would have been more comparable with clinical practice and may have resulted in a more realistic estimation of reproducibility. However, these assessments have already been performed in other WSS reproducibility studies with time intervals between first and second scan varying from 2 to 4 weeks [12] to 1 year [7].

In conclusion, reproducibility of phase-specific WSS assessment in this scan–rescan study in healthy volunteers was good for mean 3D systolic WSS for all thoracic aortic segments over multiple systolic phases. Maximum 3D systolic WSS showed more variability, up to 31% for the proximal ascending aortic region. Although intra- and interobserver reproducibility is good to excellent, these scan–rescan WSS variations should be considered to avoid misinterpretation by investigators in case of individual follow-up or in comparative rest–stress studies.

## Electronic supplementary material

Below is the link to the electronic supplementary material.

Supplementary material 1 (PDF 60 kb)

Supplementary material 2 (PDF 61 kb)

Supplementary material 3 (PDF 61 kb)

Supplementary material 4 (PDF 60 kb)

Supplementary material 5 (PDF 60 kb)

Supplementary material 6 (PDF 61 kb)

Supplementary material 7 (PDF 60 kb)

Supplementary material 8 (PDF 61 kb)
