# Supplementary material for: Scan–rescan reproducibility of segmental aortic wall shear stress as assessed by phase-specific segmentation with 4D flow MRI in healthy volunteers
Source: MAGMA. 2018 May 26;31(5):653–63. doi: 10.1007/s10334-018-0688-6 (PMC6132557; doi:10.1007/s10334-018-0688-6)
Supplement: Supplementary file 1 — Supplementary material 1 (PDF 60 kb) [file 10334_2018_688_MOESM1_ESM.pdf]

Supplementary Table 1 Intraobserver variability of segmental WSS analysis of the *peak systolic cardiac phase-2* from the scan exams

	**WSSmax (mPa)**						**WSSmean (mPa)**					
	**Bland-Altman**		**COV** (%)	**Correlation***		**ICC**	**Bland-Altman**		**COV** (%)	**Correlation***		**ICC**
	Mean difference (mPa)	*Limits of agreement (±2σ) (mPa)*		*r*	*P*		*Mean difference (mPa)*	*Limits of agreement (±2σ) (mPa)*		*r*	*P*	
**Proximal AAo**	53.2	250.0	4	0.95	<0.001	0.97	71.1	127.1	5	0.96	<0.001	0.98
**Distal AAo**	-51.7	483.3	9	0.76	0.011	0.91	5.6	53.8	2	0.99	<0.001	0.99
**Aortic arch**	-11.6	164.8	4	0.98	<0.001	0.97	-3.0	50.0	2	0.94	<0.001	0.98
**Proximal DAo**	1.2	115.9	3	0.93	<0.001	0.95	-6.1	46.9	2	0.94	<0.001	0.98
**Distal DAo**	-39.9	239.0	6	0.94	<0.001	0.89	-10.4	95.6	4	0.92	<0.001	0.94

*Spearman correlation coefficient
AAo ascending aorta, *DAo* descending aorta, *COV* coefficient of variation, *ICC* intraclass correlation coefficient

Title: Scan-rescan reproducibility of segmental aortic wall shear stress as assessed by phase-specific segmentation with 4D flow MRI in healthy volunteers
Journal: Magnetic Resonance Materials in Physics, Biology and Medicine
Authors Roel LF van der Palen, Arno AW Roest, Pieter J van den Boogaard, Albert de Roos, Nico A Blom, Jos JM Westenberg
Corresponding author: Roel LF van der Palen; Division of Pediatric Cardiology, department of Pediatrics, Leiden University Medical Center, Leiden, the Netherlands. Albinusdreef 2, 2333 ZA, Leiden, the Netherlands. E-mail: r.vanderpalen@lumc.nl
