# Supplementary material for: Scan–rescan reproducibility of segmental aortic wall shear stress as assessed by phase-specific segmentation with 4D flow MRI in healthy volunteers
Source: MAGMA. 2018 May 26;31(5):653–63. doi: 10.1007/s10334-018-0688-6 (PMC6132557; doi:10.1007/s10334-018-0688-6)
Supplement: Supplementary file 3 — Supplementary material 3 (PDF 61 kb) [file 10334_2018_688_MOESM3_ESM.pdf]

Supplementary Table 3 Intraobserver variability of segmental WSS analysis of the *peak systolic cardiac phase+1* from the scan exams

	WSSmax (mPa)						WSSmean (mPa)					
	Bland-Altman		COV (%)	Correlation*		ICC	Bland-Altman		COV (%)	Correlation*		ICC
	Mean difference (mPa)	*Limits of agreement (±2σ) (mPa)*		*r*	*P*		*Mean difference (mPa)*	*Limits of agreement (±2σ) (mPa)*		*r*	*P*	
**Proximal AAo**	111.1	327.8	8	0.92	<0.001	0.97	49.4	74.9	4	0.99	<0.001	0.99
**Distal AAo**	5.9	149.9	4	0.95	<0.001	0.98	3.2	25.4	1	0.99	<0.001	1.00
**Aortic arch**	-27.5	152.0	4	0.93	<0.001	0.98	9.5	43.6	2	0.99	<0.001	0.99
**Proximal DAo**	-15.0	148.2	3	0.96	<0.001	0.99	-0.1	88.7	3	0.95	<0.001	0.98
**Distal DAo**	-22.5	252.0	5	0.72	0.018	0.97	-24.7	86.7	3	0.99	<0.001	0.98

*Spearman correlation coefficient
AAo ascending aorta, *DAo* descending aorta, *COV* coefficient of variation, *ICC* intraclass correlation coefficient

Title: Scan-rescan reproducibility of segmental aortic wall shear stress as assessed by phase-specific segmentation with 4D flow MRI in healthy volunteers
Journal: Magnetic Resonance Materials in Physics, Biology and Medicine
Authors Roel LF van der Palen, Arno AW Roest, Pieter J van den Boogaard, Albert de Roos, Nico A Blom, Jos JM Westenberg
Corresponding author: Roel LF van der Palen; Division of Pediatric Cardiology, department of Pediatrics, Leiden University Medical Center, Leiden, the Netherlands. Albinusdreef 2, 2333 ZA, Leiden, the Netherlands. E-mail: r.vanderpalen@lumc.nl
