# Supplementary material for: Scan–rescan reproducibility of segmental aortic wall shear stress as assessed by phase-specific segmentation with 4D flow MRI in healthy volunteers
Source: MAGMA. 2018 May 26;31(5):653–63. doi: 10.1007/s10334-018-0688-6 (PMC6132557; doi:10.1007/s10334-018-0688-6)
Supplement: Supplementary file 4 — Supplementary material 4 (PDF 60 kb) [file 10334_2018_688_MOESM4_ESM.pdf]

Supplementary Table 4 Intraobserver variability of segmental WSS analysis of the *peak systolic cardiac phase+2* from the scan exams

	**WSSmax (mPa)**						**WSSmean (mPa)**					
	**Bland-Altman**		**COV** (%)	**Correlation***		**ICC**	**Bland-Altman**		**COV** (%)	**Correlation***		**ICC**
	Mean difference (mPa)	*Limits of agreement (±2σ) (mPa)*		*r*	*P*		*Mean difference (mPa)*	*Limits of agreement (±2σ) (mPa)*		*r*	*P*	
**Proximal AAo**	16.4	153.9	5	0.94	<0.001	0.99	51.5	85.8	6	1.00	<0.001	0.97
**Distal AAo**	-1.1	30.6	1	0.99	<0.001	1.00	1.6	28.9	2	0.99	<0.001	1.00
**Aortic arch**	25.1	136.6	5	0.96	<0.001	0.97	2.3	51.7	3	0.98	<0.001	0.98
**Proximal DAo**	-38.9	209.8	5	0.89	0.001	0.97	-15.6	137.8	7	0.92	<0.001	0.92
**Distal DAo**	-49.2	334.8	8	0.93	<0.001	0.94	-18.1	113.9	5	0.99	<0.001	0.96

*Spearman correlation coefficient

AAo ascending aorta, *DAo* descending aorta, *COV* coefficient of variation, *ICC* intraclass correlation coefficient

Title: Scan-rescan reproducibility of segmental aortic wall shear stress as assessed by phase-specific segmentation with 4D flow MRI in healthy volunteers
Journal: Magnetic Resonance Materials in Physics, Biology and Medicine
Authors Roel LF van der Palen, Arno AW Roest, Pieter J van den Boogaard, Albert de Roos, Nico A Blom, Jos JM Westenberg
Corresponding author: Roel LF van der Palen; Division of Pediatric Cardiology, department of Pediatrics, Leiden University Medical Center, Leiden, the Netherlands. Albinusdreef 2, 2333 ZA, Leiden, the Netherlands. E-mail: r.vanderpalen@lumc.nl
