# Supplementary material for: Scan–rescan reproducibility of segmental aortic wall shear stress as assessed by phase-specific segmentation with 4D flow MRI in healthy volunteers
Source: MAGMA. 2018 May 26;31(5):653–63. doi: 10.1007/s10334-018-0688-6 (PMC6132557; doi:10.1007/s10334-018-0688-6)
Supplement: Supplementary file 5 — Supplementary material 5 (PDF 60 kb) [file 10334_2018_688_MOESM5_ESM.pdf]

Supplementary Table 5 Interobserver variability of segmental WSS analysis of the *peak systolic cardiac phase-2* from the scan exams

	**WSSmax (mPa)**						**WSSmean (mPa)**					
	**Bland-Altman**		**COV** (%)	**Correlation***		**ICC**	**Bland-Altman**		**COV** (%)	**Correlation***		**ICC**
	Mean difference (mPa)	*Limits of agreement (±2σ) (mPa)*		*r*	*P*		*Mean difference (mPa)*	*Limits of agreement (±2σ) (mPa)*		*r*	*P*	
**Proximal AAo**	-61.9	578.1	9	0.84	0.002	0.86	-68.9	323.8	11	0.76	0.011	0.86
**Distal AAo**	-99.0	387.1	7	0.82	0.004	0.94	-1.7	48.5	2	0.99	<0.001	0.99
**Aortic arch**	-1.5	205.8	4	0.96	<0.001	0.96	21.5	74.6	4	0.95	<0.001	0.95
**Proximal DAo**	-70.2	234.2	6	0.84	0.002	0.84	-6.8	43.4	2	0.98	<0.001	0.98
**Distal DAo**	-142.4	230.7	6	0.95	<0.001	0.90	-14.3	101.5	4	0.93	<0.001	0.94

*Spearman correlation coefficient
AAo ascending aorta, *DAo* descending aorta, *COV* coefficient of variation, *ICC* intraclass correlation coefficient

Title: Scan-rescan reproducibility of segmental aortic wall shear stress as assessed by phase-specific segmentation with 4D flow MRI in healthy volunteers
Journal: Magnetic Resonance Materials in Physics, Biology and Medicine
Authors Roel LF van der Palen, Arno AW Roest, Pieter J van den Boogaard, Albert de Roos, Nico A Blom, Jos JM Westenberg
Corresponding author: Roel LF van der Palen; Division of Pediatric Cardiology, department of Pediatrics, Leiden University Medical Center, Leiden, the Netherlands. Albinusdreef 2, 2333 ZA, Leiden, the Netherlands. E-mail: r.vanderpalen@lumc.nl
