# Supplementary material for: Scan–rescan reproducibility of segmental aortic wall shear stress as assessed by phase-specific segmentation with 4D flow MRI in healthy volunteers
Source: MAGMA. 2018 May 26;31(5):653–63. doi: 10.1007/s10334-018-0688-6 (PMC6132557; doi:10.1007/s10334-018-0688-6)
Supplement: Supplementary file 8 — Supplementary material 8 (PDF 61 kb) [file 10334_2018_688_MOESM8_ESM.pdf]

Supplementary Table 8 Interobserver variability of segmental WSS analysis of the *peak systolic cardiac phase+2* from the scan exams

	WSSmax (mPa)						WSSmean (mPa)					
	**Bland-Altman**		**COV (%)**	**Correlation***		**ICC**	**Bland-Altman**		**COV (%)**	**Correlation***		**ICC**
	Mean difference (mPa)	*Limits of agreement (±2σ) (mPa)*		*r*	*P*		*Mean difference (mPa)*	*Limits of agreement (±2σ) (mPa)*		*r*	*P*	
**Proximal AAo**	-99.3	407.6	13	0.81	0.005	0.91	-29.2	120.3	7	0.89	<0.001	0.93
**Distal AAo**	-22.3	125.5	4	0.98	<0.001	0.98	7.3	45.9	3	1.0	<0.001	0.99
**Aortic arch**	42.6	153.1	5	0.94	<0.001	0.96	9.2	44.8	3	0.98	<0.001	0.98
**Proximal DAo**	-75.0	192.9	5	0.84	0.002	0.97	-30.6	125.6	6	0.92	<0.001	0.93
**Distal DAo**	-164.0	494.3	11	0.77	0.009	0.88	-36.6	84.3	3	0.95	<0.001	0.98

*Spearman correlation coefficient
AAo ascending aorta, *DAo* descending aorta, *COV* coefficient of variation, *ICC* intraclass correlation coefficient

Title: Scan-rescan reproducibility of segmental aortic wall shear stress as assessed by phase-specific segmentation with 4D flow MRI in healthy volunteers
Journal: Magnetic Resonance Materials in Physics, Biology and Medicine
Authors Roel LF van der Palen, Arno AW Roest, Pieter J van den Boogaard, Albert de Roos, Nico A Blom, Jos JM Westenberg
Corresponding author: Roel LF van der Palen; Division of Pediatric Cardiology, department of Pediatrics, Leiden University Medical Center, Leiden, the Netherlands. Albinusdreef 2, 2333 ZA, Leiden, the Netherlands. E-mail: r.vanderpalen@lumc.nl
